# Risk of Sarcopenia and Associated Factors in Older Adults with Type 2 Diabetes: An Exploratory Cross-Sectional Study

**DOI:** 10.3390/healthcare11142081

**Published:** 2023-07-21

**Authors:** Elena Massimino, Anna Izzo, Carmen Castaldo, Erica Ferretti, Angela Albarosa Rivellese, Giuseppe Della Pepa

**Affiliations:** 1 Department of Clinical Medicine and Surgery, Federico II University, Via Sergio Pansini 5, 80131 Naples, Italy; elenamassimino@libero.it (E.M.); ariannaizzo.1991@gmail.com (A.I.); carmencastaldo1998@gmail.com (C.C.); erica.ferretti@hotmail.it (E.F.); rivelles@unina.it (A.A.R.); 2 Cardiometabolic Risk Unit, Institute of Clinical Physiology, National Research Council-CNR, Via Giuseppe Moruzzi 1, 56124 Pisa, Italy

**Keywords:** risk evaluation, malnutrition, sarcopenia, type 2 diabetes, older adults

## Abstract

Background: Evidence on the risk of sarcopenia and associated factors in older adults with type 2 diabetes (T2D) is lacking. We evaluate (1) the proportion of patients at risk of sarcopenia in older adults with T2D; and (2) the factors associated with the risk of sarcopenia. Methods: We conducted a cross-sectional study on T2D patients over 65 years referred to our outpatient clinic and who carried out the yearly complication assessment visit. Eligible patients were administered questionnaires during phone interviews for the risk evaluation of sarcopenia (SARC-F), the risk evaluation of malnutrition (Mini Nutritional Assessment Short Form (MNA^®^-SF)), the adherence to the Mediterranean diet (MEDI-quest), and the evaluation of physical activity (the International Physical Activity Questionnaire short form). Results: A total of 138 patients were included in the study, and 12 patients (8.7% (95% CI 4.6–14.7)) were at risk of sarcopenia. The mean SARC-F score was significantly higher in women compared with men (2.1 ± 1.8 vs. 0.9 ± 1.4, respectively; *p* < 0.001). The majority of patients identified at risk of sarcopenia compared with those not at risk were women (75% vs. 30%, respectively; *p* = 0.003), had a higher proportion of neuropathy (50% vs. 19%, respectively; *p* = 0.027), a lower mean MNA^®^-SF score (11.6 ± 1.5 vs. 13.0 ± 1.4, respectively; *p* = 0.001), a lower mean MEDI-quest score (5.2 ± 1.5 vs. 5.9 ± 1, respectively; *p* = 0.037), and were more inactive (92% vs. 61%, respectively; *p* = 0.032). Conclusions: In a sample of older adults with T2D, the risk of sarcopenia was identified in 8.7% (95% CI: 4.6–14.7) of the sample, and the main factors associated were female gender, neuropathy, a lower MNA^®^-SF score, low adherence to the Mediterranean diet, and low physical activity.

## 1. Introduction

Sarcopenia is a pathological condition characterized by the gradual loss of skeletal muscle mass, strength, and/or function [1]. Sarcopenia is associated with an increased risk of poor outcomes such as falls, functional decline, frailty, and mortality [1].

Sarcopenia has received growing interest over the last years for the increase in life expectancy, for its global diffusion, and for its strong relationship with several chronic diseases [2], and it is now recognized as a distinct condition by an ICD-10-CM code (i.e., M 62.84) [3].

Attention has been paid to sarcopenia in individuals with type 2 diabetes (T2D) [4] due to the heavy impact that sarcopenia may have on these patients in terms of disability [5], representing a further complication of diabetes [6]. In patients with T2D, older age and a low body mass index have been shown to be significant risk factors for sarcopenia, as well as microvascular complications, i.e., retinopathy and neuropathy [4]. In terms of lifestyle factors, T2D individuals with sarcopenia have considerably lower caloric and omega-3 fatty acid intakes as well as regular physical activity than those without this condition [4].

With regard to dietary habits, the majority of research on the association between nutrition and sarcopenia has concentrated on the consumption of specific nutrients or dietary groups, mainly protein [7,8,9]. The impact of more complex dietary patterns, such as the Mediterranean diet, on sarcopenia has received less attention [10,11,12,13], and evidence linking a decreased risk of physical frailty and sarcopenic symptomology with greater adherence to the Mediterranean diet in older adults is sparse [10,14].

Sarcopenia is diagnosed by the presence of decreased muscle mass or quality, and guidelines recommend the use of dual-energy X-ray absorptiometry and/or bioelectrical impedance analysis to detect low muscle mass, and other methods such as magnetic resonance imaging and computed tomography [1]. These tools might not be useful as screening tests for the entire population of older adults, and are extremely expensive, time consuming, and unworkable approaches, particularly in underserved clinical contexts. Consequently, sarcopenia screening instruments such as questionnaires might represent a valid option.

The use of the SARC-F questionnaire—validated in community healthcare and other clinical settings [15], as well as for remote consultation [16]—could represent a valid tool recommended by the European Working Group on Sarcopenia in Older People (EWGSOP) to identify people at risk of sarcopenia [1].

The SARC-F might be useful and feasible, particularly in certain conditions, among nursing home residents, for example, or considering extreme conditions characterized by social restrictions and the reduction in medical resources. In this regard, medical remote monitoring and consultation have gained relevance, as we have learned from the last pandemic of COVID-19 and the related government restrictions imposed to counteract the pandemic, and they could be suitable for every situation in the future that might limit the availability of healthcare systems [17,18].

Currently, few studies, particularly those performed in the Asian population, have investigated the risk of sarcopenia and its associated factors, showing that a higher SARC-F score was associated with hospitalization, disability in the activities of daily living [19], sleep disorders [20], and mild cognitive impairment [21] in older adults with T2D. Scant evidence is available on the European population, in which the risk of sarcopenia has been associated with female gender and respiratory and neurological diseases in older adults without T2D [22,23], and no data, so far, are available with regard to Italian individuals.

Against this background, the aim of this exploratory cross-sectional study was to evaluate: (1) the proportion of patients at risk of sarcopenia; and (2) the associated factors (i.e., clinical parameters, lifestyle factors, adherence to the Mediterranean diet) in a sample of older adults with T2D, followed in a diabetes outpatient clinic in southern Italy, in order to provide evidence for possible prevention and therapeutic approaches.

## 2. Materials and Methods

### 2.1. Study Design and Participants

We conducted a cross-sectional study on T2D patients aged over 65 who underwent the yearly complication assessment visit at the Diabetes Unit of Federico II University of Naples from June 2020 to September 2020. In the study, patients with T2D who were 65 years old and over and able to answer the study questionnaires during phone interviews were included. The exclusion criteria were the following: eating disorders, chronic diabetic complications that potentially impact food intake (such as gastroparesis and atrophic gastritis), history of alcoholism and substance abuse, acute or chronic diseases that significantly impair health status, and conditions limiting data collection. Of the 217 patients with T2D who underwent the yearly complication assessment visit, 154 were older adults meeting the inclusion criteria; these patients were administered, between April 2021 and June 2021, questionnaires during phone interviews in order to evaluate the risk for sarcopenia and malnutrition, dietary habits and adherence to the Mediterranean diet, and the evaluation of physical activity.

The Ethics Committee of Federico II University approved the study protocol, which was performed in accordance with the Declaration of Helsinki, and each participant provided written informed consent to the use of their clinical and laboratory data, and to being included in the study.

### 2.2. Sample Size

The primary aim was to determine the proportion of patients at risk of sarcopenia in an Italian sample of older adults with T2D. As there has been no study on the risk of sarcopenia in older patients with T2D in Italy, we used the prevalence estimate (7.5%) for sarcopenia among older adults without T2D, and with similar characteristics to our sample, from an Italian survey [24]. The sample size was computed to be 107, at 5% precision and 95% confidence level, and used the following sample size formula: *n* = Z^2^ × P × (1 − P)/d^2^, where Z is the Z statistic for a level of confidence, P is the expected prevalence, and d is the precision level.

### 2.3. Outcomes

The study outcomes included the proportion of patients at risk of sarcopenia according to the SARC-F, and the evaluation of anthropometric, clinical, and nutritional factors, including adherence to the Mediterranean diet, associated with the risk of sarcopenia.

### 2.4. Assessment of Clinical Parameters

The clinical parameters and screening for chronic complications of diabetes were evaluated during the yearly complication assessment visit from June 2020 to September 2020.

The assessment of chronic complications, according to a standardized protocol, included a dilated eye exam for the detection of diabetic retinopathy; serum creatinine, eGFR, and urine albumin excretion rate, which were used to evaluate nephropathy; standardized cardiovascular reflex tests, used to examine the function of the autonomic nervous system; parasympathetic and sympathetic nervous system activity, which were each measured by measuring the variability of the heart rate during a deep breathing test (also known as the “beat-to-beat variation test”); and an ankle reflex test, a Semmes–Weinstein monofilament tactile perception test, and a bilateral vibration perception test, which were used to detect peripheral neuropathy.

Smoking status was defined as smoking one or more cigarettes per day. Alcohol consumption was defined as a dichotomic variable (drinkers and non-drinkers).

Data on weight, body mass index (BMI), comorbidities, and drugs taken were collected from the medical records (data collected within 2–4 months prior to the phone interviews). Height was determined by a bar-altimeter, waist circumference by an elastic meter, and body weight by a mechanic balance (Seca 709). All measurements were taken with accuracy to the nearest 0.1 kg and 0.1 cm, with the patient in light clothing and no shoes. BMI was calculated as body weight in kilograms divided by the square of body height in meters.

### 2.5. Risk Evaluation for Sarcopenia

The risk evaluation for sarcopenia was assessed using the SARC-F, a five-item self-report screening questionnaire [15]. The questionnaire evaluated the difficulties that participants might have in performing some activities able to assess strength such as assistance in walking, rising from a chair, and climbing stairs. For each item, there were three possible answers—not problematic at all, somewhat problematic, and extremely problematic or unable to do—that were awarded 0, 1, and 2 points, respectively. The last item regarded the number of falls and is determined by asking participants how many times they have fallen in the past year; 0 falls are scored 0, 1–3 falls are scored 1, and 4 or more falls are scored 2. The total score ranges from 0 to 10, and the risk of sarcopenia is identified by a score ≥4 [15].

### 2.6. Risk Evaluation for Malnutrition

The risk evaluation for malnutrition was assessed using the MNA^®^-SF questionnaire consisting of the assessment of anthropometric parameters (BMI, weight loss), questions on global assessment (mobility), dietary habit evaluation (food intake), and health assessment (neuropsychological problems and acute disease). For each question, there is a score ranging from 0 to 3. A total score ≥12, between 11–8, and ≤7 identifies no malnutrition, risk of malnutrition, and malnutrition, respectively [25,26].

### 2.7. Assessment of Dietary Habits and Adherence to the Mediterranean Diet

Dietary intake data were assessed by three 24-hour recalls (collected by phone interviews on two weekdays and on one weekend day) and calculated using the nutritional software MetaDieta (Meteda s.r.l., Ascoli-Piceno). The adherence to the Mediterranean diet was assessed using the MEDI-quest score [27], a food frequency questionnaire that considers nine food categories: wholegrain cereals, legumes and nuts, fruit, vegetables, meat, fish, foods rich in animal fat such as butter, cake, pastries, dairy products (not milk and yogurt), alcohol intake, and olive oil. For fruit, vegetables, wholegrain cereals, legumes and nuts, and fish (food groups typical of the Mediterranean diet), 1 point was assigned to the highest category of consumption, 0.5 to the middle category, and 0 to the lowest category. On the other hand, for meat and foods rich in animal fat (food groups not typical of the Mediterranean diet), 1 point was assigned to the lowest category, 0.5 to the middle category, and 0 points for the highest category of consumption.

For alcohol, 1 point was given for 1–2 glasses of wine/day, 0.5 points for 4 glasses of wine/day, and 0 to those who did not drink alcohol. Finally, for olive oil, 1 point was given for daily use, 0.5 point for 4–6 times a week, and 0 points for <4 times a week.

According to the scoring system of the MEDI-quest, four possible categories can be identified: 0–3 (low adherence), 4–5 (unsatisfactory adherence), 6–7 (high adherence), and 8–9 (very high adherence) [27].

### 2.8. Assessment of Physical Activity

The physical activity of the patients was assessed by the International Physical Activity Questionnaire short form (IPAQ-SF) [28], and according to the scoring system by the IPAQ scoring protocol, three possible categories can be classified: inactive (<700 MET × week), moderately active (700–2500 MET × week), and active (>2500 MET × week), according to the scoring [28].

### 2.9. Statistical Analysis

Data are presented as mean ± standard deviation for continuous variables or frequencies and percentages for categorical variables. The anthropometric and clinical parameters of the participants, as well as nutritional and lifestyle factors, were compared according to the risk of sarcopenia (not at risk/at risk) using an independent sample t-test for normally distributed continuous data, and a Mann–Whitney U test for non-normally distributed continuous data. The Fisher exact test was used for categorical variables. A *p*-value < 0.05 was considered statistically significant. Statistical analyses were performed using SPSS 26.0 software (SPSS/PC; IBM, Armonk, NY, USA).

## 3. Results

### 3.1. Characteristics of Participants and Risk of Sarcopenia

A total of 154 older adults with T2D met the inclusion criteria; of these, 16 were excluded for missing data. A total of 138 (34% women) older patients with T2D were included in the study. Their clinical and anthropometric characteristics are shown in Table 1, as total sample and divided by gender. Women had a significantly higher BMI (*p* = 0.001), lower body weight (*p* = 0.036), and consumed less alcohol (*p* = 0.042).

The mean SARC-F score in our sample was 1.3 ± 1.7, and it was significantly higher in women compared with men (2.1 ± 1.8 vs. 0.9 ± 1.4, respectively; *p* < 0.001) (Table 1).

In total 12 patients out of 138, 8.7% (95% CI: 4.6–14.7) of the sample, had a SARC-F score ≥4 and were defined as at risk of sarcopenia. The patients at risk of sarcopenia compared with those not at risk were significantly different according to gender; in fact, the majority of the patients identified as being at risk of sarcopenia compared with those not at risk were women (75% (9 out of 12) vs. 30% (38 out of 126), respectively; *p* = 0.003). The proportion of men and women identified in the sample as being at risk of sarcopenia was 3.3% (95% CI: 0.7–9.3) and 19.1% (95% CI: 9.1–33.3), respectively.

Patients at risk of sarcopenia compared with those not at risk had a significantly higher proportion of neuropathy (50% vs. 19%, respectively; *p* = 0.027); no other significant differences were observed with regard to anthropometric and metabolic parameters among the groups (Table 2).

### 3.2. Dietary Habits, Risk of Malnutrition, Adherence to Mediterranean Diet, and Physical Activity according to the Risk of Sarcopenia

The dietary habits of the whole sample show a low protein intake for ideal body weight (0.9 ± 0.2 g/kg/day) compared to recommendations for older adults (1.0–1.2 g/kg/day [29]) (Table 3).

Although there were no differences in the dietary habits between patients at risk and not at risk of sarcopenia, the mean MNA^®^-SF score in the patients at risk of sarcopenia was significantly lower compared with patients not at risk of sarcopenia (11.6 ± 1.5 vs. 13.0 ± 1.4, respectively; *p* = 0.001) (Table 3). According to the MNA^®^-SF score, a normal nutritional status was observed in 113 (82%) patients, while 25 (18%) were at risk of malnutrition, and none was malnourished.

Data from the MEDI-quest show that the patients at risk of sarcopenia compared with those not at risk had a significantly lower mean score (5.2 ± 1.5 vs. 5.9 ± 1, respectively; *p* = 0.037). The majority of our sample was inactive (64%), and the patients at risk of sarcopenia were significantly more inactive (92% vs. 61%, respectively; *p* = 0.032) (Table 3).

## 4. Discussion

In the present exploratory cross-sectional study, we have shown that in a sample of older adults with T2D from a single diabetes outpatient clinic in southern Italy, the risk of sarcopenia was identified in 12 patients out of 138, 8.7% (95% CI: 4.6–14.7) of the sample, and the main factors associated with the risk of sarcopenia were female gender, neuropathy, a lower MNA^®^-SF score, low adherence to the Mediterranean diet, and low physical activity.

Many epidemiological studies, particularly those performed in Asian populations, have shown that genetic factors, gender, ethnic background, and modifiable variables, including malnutrition, socioeconomic status, physical inactivity, smoking, comorbidities, hospitalization, and medications, are likely contributors to the development of sarcopenia [30,31]. Evidence on the prevalence of older adults at risk for sarcopenia and associated factors in non-Asian countries is scarce [4].

In our study, older adults with T2D at risk of sarcopenia were 8.7% (95% CI: 4.6–14.7), and the majority of the patients identified as being at risk of sarcopenia were women (75%, 9 out 12). The proportion of men and women identified in the sample as being at risk of sarcopenia was 3.3% (95% CI: 0.7–9.3) and 19.1% (95% CI: 9.1–33.3), respectively.

The few studies evaluating the risk of sarcopenia according to the SARC-F in patients with diabetes have shown a prevalence ranging from 19% to 29% [19,20,21] in Asian or mixed American populations, with a higher prevalence in women compared with men (~26% vs. ~17%, respectively) [20,21]. In the European population, Milewska et al. identified a prevalence of the risk of sarcopenia in Polish older adults that doubled compared with our data (18.6%), with a higher prevalence in women compared with men (22.3% vs. 13.2%, respectively) [22]. Similar prevalence (10%–33%) was reported by Krzyminska-Siemaszko et al., who identified a trend toward more frequent prevalence in women than in men (19.8% vs. 11.8%, respectively) [23].

It is important to underline that our study is the first performed in Italy to detect the risk of sarcopenia using SARC-F in older adults with T2D, at least to our knowledge. Thus, we cannot make any comparison, although our data seem to be in line with those found in Italian older adults without T2D showing a risk of sarcopenia ranging from 7.5% to 9% [24,32]. The lower prevalence observed in our study might be related to the exclusion of patients with chronic diseases severely affecting their health status and those with mobility difficulties.

According to the last published algorithm, the SARC-F is recommended as the first screening tool to identify individuals at risk of sarcopenia [1,33], and it has been validated for remote consultation [16]. This aspect is of paramount relevance considering the impact of the last COVID-19 pandemic and the government’s restrictions imposed to contain the pandemic, and it can be appropriate for every circumstance that might restrict access to the healthcare system in the future [34].

The SARC-F has also been validated in older adults with T2D [35], showing a high specificity (men 85.8%, women 72.4%), low sensitivity (men 14.6%, women 33.3%), and low negative predictive value (men 67.5%, women 86.2%) when the diagnostic criteria for sarcopenia based on the EWGSOP guidelines were assumed as a reference [35]. Despite its low sensitivity, through the identification of patients at risk of sarcopenia by short and structured telephone interviews with the SARC-F, it is possible to select patients for whom further tests might be performed in presence.

In our sample, the proportion of neuropathy was higher in patients at risk of sarcopenia (50% vs. 19%, respectively), in line with current evidence showing a strong association between diabetic neuropathy and sarcopenia with a pooled odds ratio of 1.62 (95% CI: 1.30–2.02) [36]. Various pathophysiological mechanisms have been proposed to explain the relationship between peripheral neuropathy and the risk of sarcopenia in patients with T2D, such as blood glucose control, an increase in advanced glycosylation end products, oxidative stress, inflammation, and loss of motor units [7]. The early identification of neuropathy in older adults with T2D might represent a further strategy to prevent and delay sarcopenia.

In our sample, a lower MNA^®^-SF score was another factor associated with the risk of sarcopenia. In this regard, sarcopenia and malnutrition are strongly associated, and many patients present both conditions, configuring the malnutrition–sarcopenia syndrome [37,38,39]. Growing evidence shows that malnutrition is extremely prevalent in older adults [40,41], representing a major burden responsible for many pathological conditions [42,43]. Furthermore, malnourished older adults are at risk of sarcopenia [37,44,45], and evidence shows that older adults at risk of malnutrition have a higher risk of sarcopenia (OR = 13.6; 95% CI: 1.55–11.38) [46]. Importantly, our data suggest that even a minimal abnormality, as indicated by a small reduction in the MNA^®^-SF score, may represent a risk factor for sarcopenia.

The risk of malnutrition was identified in 18% of the sample. Several studies have estimated a 31%–55% risk of malnutrition in older patients with diabetes [47,48,49,50,51]. The different prevalence could be explained by several factors, such as ethnicity, living, or clinical conditions that might affect nutritional status. Considering more in-depth dietary habits, the whole sample, independently of the risk of sarcopenia, had an inadequate protein intake for ideal body weight (0.9 ± 0.2 g/kg/day) compared to the amount recommended for older adults (1.0–1.2 g/kg/day) and for older adults who are malnourished or at risk of malnutrition (1.2–1.5 g/kg/day) [29]. Several studies suggest that sarcopenia is associated with inadequate dietary proteins, and a low protein intake is associated with lower muscle mass [50,51,52].

Beyond single nutrient intake, research into the effectiveness of dietary patterns on age-related health outcomes has become increasingly relevant, suggesting that overall diet quality is linked to a lower risk of age-related diseases [53]. In this regard, in our study, the risk of sarcopenia was also associated with lower adherence to the Mediterranean diet, in line with other studies reporting a significant association between Mediterranean diet adherence and decreased risk of sarcopenia [14,54]. Of note, in the Mediterranean countries, a progressive shift towards more Western dietary models is also occurring in older adults [55], and adherence to the Mediterranean model should be encouraged in this population. Including more traditional foods characterizing the Mediterranean diet, i.e., fruits and vegetables, legumes, whole grains, extra virgin olive oil, fish, and dairy products, has been shown to be particularly effective at restoring the recommended levels of vitamins, antioxidants, polyunsaturated fatty acids, and fiber [55], which might mitigate the factors associated with age-related muscle strength decline, including inflammation, endothelial dysfunction, oxidative stress, and insulin resistance [10].

In our study, low physical activity was associated with the risk of sarcopenia; notably, a high proportion of the sample was inactive (60% of the whole sample). Our findings support the existing evidence available and suggest that the practice of physical activity is a promising intervention to prevent and delay sarcopenia through well-known beneficial effects such as the increase in muscle strength and mass, and the prevention of many chronic diseases favoring sarcopenia [56]. Indeed, a meta-analysis of 25 studies revealed that physical activity was associated with a significant decrease in the odds of sarcopenia (OR = 0.45, 95% CI: 0.37–0.55) [57].

This study has some strengths, such as the use of validated tools to evaluate the risk of both sarcopenia (SARC-F) and malnutrition (MNA^®^-SF), and the availability of a medical record for all patients. However, some limitations should be reported: the small sample size, and the corresponding small number of men and women identified at risk of sarcopenia; the low sensitivity of the SARC-F; the cross-sectional nature of the study, which does not allow the identification of cause–effect relationships; the fact that data from a single study site would not reflect the nationwide risk of sarcopenia; and our data, which refer to a single study centre of southern Italy, which may not be representative of the general population of older adults with T2D. Furthermore, we excluded patients with chronic disease severely affecting their health status and with mobility difficulties; this might have resulted in an underestimation of patients at risk of sarcopenia. Finally, the limitations of the phone interview should be considered. However, previous studies showed that a well-structured and expertly managed 24 h dietary recall by phone can provide an accurate estimate of macronutrient intake [58].

Active monitoring and remote care system evaluation might have a beneficial impact on older adults with T2D living in self-isolation, promoting the achievement of optimal physical and psychological well-being [59]. The identification of risk factors associated with the risk of sarcopenia might suggest possible strategies for preventing sarcopenia in older adults with T2D, such as promoting nutritional intake, adherence to the Mediterranean diet, and physical activity, particularly in women. Furthermore, these patients will be more likely to need care in the future, favoring the design of individual approaches able to prevent further aggravation of sarcopenia and counteract the effects of isolation and social distancing imposed, for example, by pandemics.

## 5. Conclusions

In conclusion, in a sample of 138 older adults with T2D, the risk of sarcopenia was identified in 12 patients, 8.7% (95% CI: 4.6–14.7) of the sample, and the main factors associated were female gender, neuropathy, a lower MNA^®^-SF score, low adherence to the Mediterranean diet, and low physical activity. According to the results of our study, a screening test performed in remote consultation by short, structured telephone interviews for the risk evaluation of sarcopenia and associated factors should be implemented, followed by regular assessment in clinical practice to prevent or delay these deleterious conditions, and by the implementation of adequate approaches focused particularly on promoting adherence to dietary recommendations and regular physical activity. Future epidemiological studies, using a larger sample size and considering an SARC-F version with a more suitable sensitivity for older adults with T2D, are needed to confirm the trends found in our study.

## Figures and Tables

**Table 1:** Main characteristics of the study sample.

Variables	All Patients (*n* = 138)	Men (*n* = 91)	Women (*n* = 47)	*p*
Age (years)	72 ± 4	72 ± 5	72 ± 4	0.814
Body weight (Kg)	81 ± 13	82 ± 12	78 ± 14	0.036
BMI (Kg/m^2^)	29 ± 5	28 ± 4	31 ± 6	0.001
Waist circumference (cm)	102 ± 11	102 ± 10	102 ± 13	0.821
Duration of diabetes (years)	18 ± 9	18 ± 9	18 ± 11	0.612
Alcohol consumption (*n*, %)	85 (62)	62 (67)	23 (49)	0.042
Smoker (*n*, %)	33 (24)	23 (25)	10 (21)	0.678
SARC-F score (points)	1.3 ± 1.7	0.9 ± 1.4	2.1 ± 1.8	<0.001

Data are expressed as the mean ± standard deviation (SD), number (%). BMI: body mass index.

**Table 2:** Anthropometric and clinical characteristics of the study sample according to the risk of sarcopenia.

Variables	Not at Risk of Sarcopenia (*n* = 126)	At Risk of Sarcopenia (*n* = 12)	*p*
Women (n, %)	38 (30)	9 (75)	0.003
Age (years)	72 ± 5	72 ± 4	0.954
Body weight (Kg)	81 ± 12	75 ± 13	0.085
BMI (Kg/m^2^)	29 ± 5	29 ± 5	0.975
Waist circumference (cm)	102 ± 11	100 ± 12	0.508
Duration of diabetes (years)	18 ± 9	21 ± 10	0.294
Alcohol consumption (*n*, %)	80 (63)	5 (47)	0.231
Smoker (*n*, %)	29 (23)	4 (33)	0.708
Glucose-lowering drugs (*n*, %)	115 (91)	12 (100)	1.000
Rapid-acting insulin (*n*, %)	30 (29)	5 (47)	0.356
Long-acting insulin (*n*, %)	53 (42)	4 (33)	0.834
Antihypertensive drugs (*n*, %)	109 (87)	10 (83)	0.510
Lipid-lowering drugs (*n*, %)	107 (85)	10 (83)	0.602
Retinopathy (%)	25 (20)	2 (17)	1.000
Neuropathy (%)	24 (19)	6 (50)	0.027
Nephropathy (%)	31 (25)	2 (17)	0.719
Cardiovascular diseases (%)	47 (37)	2 (17)	0.211

Data are expressed as the mean ± standard deviation (SD), number (%). BMI: body mass index.

**Table 3:** Daily energy and macronutrient intakes, MNA^®^-SF score, MEDI-Quest score, and International Physical Activity Questionnaire Short Form score of the study sample in all patients and according to the risk of sarcopenia.

Variables	All Patients (*n* = 138)	Not at Risk of Sarcopenia (*n* = 126)	At Risk of Sarcopenia (*n* = 12)	*p*
Energy (Kcal/day)	1414 ± 342	1423 ± 345	1319 ± 295	0.314
Protein (g/kg)	0.9 ± 0.2	0.9 ± 0.2	0.9 ± 0.2	0.855
Protein (%TEI)	17 ± 3	17 ± 3	17 ± 3	0.920
Fat (%TEI)	35 ± 7	35 ± 6	34 ± 8	0.813
Carbohydrates (% TEI)	48 ± 7	47 ± 7	48 ± 10	0.835
Simple sugars (%TEI)	16 ± 5	16 ± 6	17 ± 5	0.455
Fibre (g/day)	16 ± 6	16 ± 6	16 ± 7	0.897
MNA^®^-SF score	12.9 ± 1.4	13.0 ± 1.4	11.6 ± 1.5	0.001
MEDI-Quest score	5.8 ± 1	5.9 ± 1	5.2 ± 1.5	0.037
IPAQ-SF score, inactive ^‡^ (*n*, %)	88 (64)	77 (61)	11 (92)	0.032

Data are expressed as the mean ± standard deviation (SD), number (%). TEI: Total Energy Intake; MNA^®^-SF: Mini Nutrition Assessment Short Form. IPAQ-SF: International Physical Activity Short Form. ^‡^ Inactive: <700 MET/week.

## Data Availability

Not applicable.

## References

[1] Cruz-Jentoft A.J., Bahat G., Bauer J., Boirie Y., Bruyère O., Cederholm T., Cooper C., Landi F., Rolland Y., Sayer A.A. (2019). Sarcopenia: Revised European consensus on definition and diagnosis. Age Ageing.

[2] Pacifico J., Geerlings M.A.J., Reijnierse E.M., Phassouliotis C., Lim W.K., Maier A.B. (2020). Prevalence of sarcopenia as a comorbid disease: A systematic review and meta-analysis. Exp. Gerontol..

[3] Cao L., Morley J.E. (2016). Sarcopenia Is Recognized as an Independent Condition by an International Classification of Disease, Tenth Revision, Clinical Modification (ICD-10-CM) Code. J. Am. Med. Dir. Assoc..

[4] Izzo A., Massimino E., Riccardi G., Della Pepa G. (2021). A Narrative Review on Sarcopenia in Type 2 Diabetes Mellitus: Prevalence and Associated Factors. Nutrients.

[5] Wong E., Backholer K., Gearon E., Harding J., Freak-Poli R., Stevenson C., Peeters A. (2013). Diabetes and risk of physical disability in adults: A systematic review and meta-analysis. Lancet Diabetes Endocrinol..

[6] Cobo A., Vázquez L.A., Reviriego J., Rodríguez-Mañas L. (2016). Impact of frailty in older patients with diabetes mellitus: An overview. Endocrinol. Nutr..

[7] Massimino E., Izzo A., Riccardi G., Della Pepa G. (2021). The Impact of Glucose-Lowering Drugs on Sarcopenia in Type 2 Diabetes: Current Evidence and Underlying Mechanisms. Cells.

[8] Calvani R., Miccheli A., Landi F., Bossola M., Cesari M., Leeuwenburgh C., Sieber C.C., Bernabei R., Marzetti E. (2013). Current nutritional recommendations and novel dietary strategies to manage sarcopenia. J. Frailty Aging.

[9] Gielen E., Beckwée D., Delaere A., De Breucker S., Vandewoude M., Bautmans I. (2021). Sarcopenia Guidelines Development Group of the Belgian Society of Gerontology and Geriatrics (BSGG). Nutritional interventions to improve muscle mass, muscle strength, and physical performance in older people: An umbrella review of systematic reviews and meta-analyses. Nutr. Rev..

[10] Cacciatore S., Calvani R., Marzetti E., Picca A., Coelho-Júnior H.J., Martone A.M., Massaro C., Tosato M., Landi F. (2023). Low Adherence to Mediterranean Diet Is Associated with Probable Sarcopenia in Community-Dwelling Older Adults: Results from the Longevity Check-Up (Lookup) 7+ Project. Nutrients.

[11] Coelho-Júnior H.J., Trichopoulou A., Panza F. (2021). Cross-sectional and longitudinal associations between adherence to Mediterranean diet with physical performance and cognitive function in older adults: A systematic review and meta-analysis. Ageing Res. Rev..

[12] Silva R., Pizato N., da Mata F., Figueiredo A., Ito M., Pereira M.G. (2018). Mediterranean Diet and Musculoskeletal-Functional Outcomes in Community-Dwelling Older People: A Systematic Review and Meta-Analysis. J. Nutr. Health Aging.

[13] Granic A., Sayer A.A., Robinson S.M. (2019). Dietary Patterns, Skeletal Muscle Health, and Sarcopenia in Older Adults. Nutrients.

[14] McClure R., Villani A. (2017). Mediterranean Diet attenuates risk of frailty and sarcopenia: New insights and future directions. J. Cachexia Sarcopenia Muscle.

[15] Malmstrom T.K., Morley J.E. (2013). SARC-F: A simple questionnaire to rapidly diagnose sarcopenia. J. Am. Med. Dir. Assoc..

[16] Krznarić Ž., Bender D.V., Laviano A., Cuerda C., Landi F., Monteiro R., Pirlich M., Barazzoni R. (2020). A simple remote nutritional screening tool and practical guidance for nutritional care in primary practice during the COVID-19 pandemic. Clin. Nutr..

[17] Lekamwasam R., Lekamwasam S. (2020). Effects of COVID-19 Pandemic on Health and Wellbeing of Older People: A Comprehensive Review. Ann. Geriatr. Med. Res..

[18] Kirwan R., McCullough D., Butler T., Perez de Heredia F., Davies I.G., Stewart C. (2020). Sarcopenia during COVID-19 lockdown restrictions: Long-term health effects of short-term muscle loss. Geroscience.

[19] Liccini A., Malmstrom T.K. (2016). Frailty and Sarcopenia as Predictors of Adverse Health Outcomes in Persons with Diabetes Mellitus. J. Am. Med. Dir. Assoc..

[20] Ida S., Kaneko R., Nagata H., Noguchi Y., Araki Y., Nakai M., Ito S., Ishihara Y., Imataka K., Murata K. (2019). Association between sarcopenia and sleep disorder in older patients with diabetes. Geriatr. Gerontol. Int..

[21] Ida S., Nakai M., Ito S., Ishihara Y., Imataka K., Uchida A., Monguchi K., Kaneko R., Fujiwara R., Takahashi H. (2017). Association Between Sarcopenia and Mild Cognitive Impairment Using the Japanese Version of the SARC-F in Elderly Patients with Diabetes. J. Am. Med. Dir. Assoc..

[22] Milewska M., Przekop Z., Szostak-Węgierek D., Chrzanowska M., Raciborski F., Traczyk I., Sińska B.I., Samoliński B. (2022). Prevalence of Risk of Sarcopenia in Polish Elderly Population-A Population Study. Nutrients.

[23] Krzymińska-Siemaszko R., Deskur-Śmielecka E., Kaluźniak-Szymanowska A., Lewandowicz M., Wieczorowska-Tobis K. (2020). Comparison of Diagnostic Performance of SARC-F and Its Two Modified Versions (SARC-CalF and SARC-F+EBM) in Community-Dwelling Older Adults from Poland. Clin. Interv. Aging.

[24] Volpato S., Bianchi L., Cherubini A., Landi F., Maggio M., Savino E., Bandinelli S., Ceda G.P., Guralnik J.M., Zuliani G. (2014). Prevalence and clinical correlates of sarcopenia in community-dwelling older people: Application of the EWGSOP definition and diagnostic algorithm. J. Gerontol. A Biol. Sci. Med. Sci..

[25] Rubenstein L.Z., Harker J.O., Salvà A., Guigoz Y., Vellas B. (2001). Screening for Undernutrition in Geriatric Practice: Developing the Short-Form Mini-Nutritional Assessment (*MNA-SF)*. J. Gerontol..

[26] Vellas B., Villars H., Abellan G., Soto M.E., Rolland Y., Guigoz Y., Morley J.E., Chumlea W., Salva A., Rubenstein L.Z. (2006). Overview of the MNA—Its history and challenges. J. Nutr. Health Aging.

[27] Vitale M., Racca E., Izzo A., Giacco A., Parente E., Riccardi G., Giacco R. (2019). Adherence to the traditional Mediterranean diet in a population of South of Italy: Factors involved and proposal of an educational field-based survey tool. Int. J. Food Sci. Nutr..

[28] Craig C.L., Marshall A.L., Sjöström M., Bauman A.E., Booth M.L., Ainsworth B.E., Pratt M., Ekelund U., Yngve A., Sallis J.F. (2003). International physical activity questionnaire: 12-country reliability and validity. Med. Sci. Sports Exerc..

[29] Volkert D., Beck A.M., Cederholm T., Cruz-Jentoft A., Goisser S., Hooper L., Kiesswetter E., Maggio M., Raynaud-Simon A., Sieber C.C. (2019). ESPEN guideline on clinical nutrition and hydration in geriatrics. Clin. Nutr..

[30] Cruz-Jentoft A.J., Sayer A.A. (2019). Sarcopenia. Lancet.

[31] Yuan S., Larsson S.C. (2023). Epidemiology of sarcopenia: Prevalence, risk factors, and consequences. Metabolism.

[32] Barnaba L., Ciarapica D., Zaccaria M., Polito A. (2017). Sarcopenia in a healthy Italian elderly population. Nutr. Met. Insights.

[33] Chen L.K., Woo J., Assantachai P., Auyeung T.W., Chou M.Y., Iijima K., Jang H.C., Kang L., Kim M., Kim S. (2020). Asian Working Group for Sarcopenia: 2019 Consensus Update on Sarcopenia Diagnosis and Treatment. J. Am. Med. Dir. Assoc..

[34] Wilder-Smith A., Freedman D.O. (2020). Isolation, quarantine, social distancing and community containment: Pivotal role for old-style public health measures in the novel coronavirus (2019-nCoV) outbreak. J. Travel. Med..

[35] Ida S., Murata K., Nakadachi D., Ishihara Y., Imataka K., Uchida A., Monguchi K., Kaneko R., Fujiwara R., Takahashi H. (2017). Development of a Japanese version of the SARC-F for diabetic patients: An examination of reliability and validity. Aging Clin. Exp. Res..

[36] Wannarong T., Sukpornchairak P., Naweera W., Geiger C.D., Ungprasert P. (2022). Association between diabetic peripheral neuropathy and sarcopenia: A systematic review and meta-analysis. Geriatr. Gerontol. Int..

[37] Vandewoude M.F., Alish C.J., Sauer A.C., Hegazi R.A. (2012). Malnutrition-sarcopenia syndrome: Is this the future of nutrition screening and assessment for older adults?. J. Aging Res..

[38] Verstraeten L.M.G., van Wijngaarden J.P., Pacifico J., Reijnierse E.M., Meskers C.G.M., Maier A.B. (2021). Association between malnutrition and stages of sarcopenia in geriatric rehabilitation inpatients: RESORT. Clin. Nutr..

[39] Faxén-Irving G., Luiking Y., Grönstedt H., Franzén E., Seiger Å., Vikström S., Wimo A., Boström A.M., Cederholm T. (2021). Do Malnutrition, Sarcopenia and Frailty Overlap in Nursing-Home Residents?. J. Frailty Aging.

[40] Leij-Halfwerk S., Verwijs M.H., van Houdt S., Borkent J.W., Guaitoli P.R., Pelgrim T., Heymans M.W., Power L., Visser M., Corish C.A. (2019). Prevalence of protein-energy malnutrition risk in European older adults in community, residential and hospital settings, according to 22 malnutrition screening tools validated for use in adults ≥65 years: A systematic review and meta-analysis. Maturitas.

[41] Cereda E., Pedrolli C., Klersy C., Bonardi C., Quarleri L., Cappello S., Turri A., Rondanelli M., Caccialanza R. (2016). Nutritional status in older persons according to healthcare setting: A systematic review and meta-analysis of prevalence data using MNA^®^. Clin. Nutr..

[42] Saunders J., Smith T. (2010). Malnutrition: Causes and consequences. Clin. Med..

[43] Beaudart C., Sanchez-Rodriguez D., Locquet M., Reginster J.Y., Lengelé L., Bruyère O. (2019). Malnutrition as a Strong Predictor of the Onset of Sarcopenia. Nutrients.

[44] Liguori I., Curcio F., Russo G., Cellurale M., Aran L., Bulli G., Della-Morte D., Gargiulo G., Testa G., Cacciatore F. (2018). Risk of Malnutrition Evaluated by Mini Nutritional Assessment and Sarcopenia in Noninstitutionalized Elderly People. Nutr. Clin. Pract..

[45] Simsek H., Meseri R., Sahin S., Kilavuz A., Bicakli D.H., Uyar M., Savas S., Sarac F., Akcicek F. (2019). Prevalence of sarcopenia and related factors in community-dwelling elderly individuals. Saudi Med. J..

[46] Sato P.H.R., Ferreira A.A., Rosado E.L. (2020). The prevalence and risk factors for sarcopenia in older adults and long-living older adults. Arch. Gerontol. Geriatr..

[47] Liu G.X., Chen Y., Yang Y.X., Yang K., Liang J., Wang S., Gan H.T. (2017). Pilot study of the Mini Nutritional Assessment on predicting outcomes in older adults with type 2 diabetes. Geriatr. Gerontol. Int..

[48] Sanz París A., García J.M., Gómez-Candela C., Burgos R., Martín Á., Matía P., Study VIDA group (2013). Malnutrition prevalence in hospitalized elderly diabetic patients. Nutr. Hosp..

[49] Martín A., Ruiz E., Sanz A., García J.M., Gómez-Candela C., Burgos R., Matía P., Ramalle-Gomera E. (2016). Accuracy of Different Mini Nutritional Assessment Reduced Forms to Evaluate the Nutritional Status of Elderly Hospitalised Diabetic Patients. J. Nutr. Health Aging.

[50] Hengeveld L.M., Boer J.M.A., Gaudreau P., Heymans M.W., Jagger C., Mendonça N., Ocké M.C., Presse N., Sette S., Simonsick E.M. (2020). Prevalence of protein intake below recommended in community-dwelling older adults: A meta-analysis across cohorts from the PROMISS consortium. J. Cachexia Sarcopenia Muscle.

[51] Smeuninx B., Greig C.A., Breen L. (2020). Amount, Source and Pattern of Dietary Protein Intake Across the Adult Lifespan: A Cross-Sectional Study. Front. Nutr..

[52] Dorhout B.G., Overdevest E., Tieland M., Nicolaou M., Weijs P.J.M., Snijder M.B., Peters R.J.G., van Valkengoed I.G.M., Haveman-Nies A., de Groot L.C.P.G.M. (2020). Sarcopenia and its relation to protein intake across older ethnic populations in the Netherlands: The HELIUS study. Ethn. Health.

[53] Milte C.M., McNaughton S.A. (2016). Dietary patterns and successful ageing: A systematic review. Eur. J. Nutr..

[54] Capurso C., Bellanti F., Lo Buglio A., Vendemiale G. (2019). The Mediterranean Diet Slows Down the Progression of Aging and Helps to Prevent the Onset of Frailty: A Narrative Review. Nutrients.

[55] Obeid C.A., Gubbels J.S., Jaalouk D., Kremers S.P.J., Oenema A. (2022). Adherence to the Mediterranean diet among adults in Mediterranean countries: A systematic literature review. Eur. J. Nutr..

[56] Yoo S.Z., No M.H., Heo J.W., Park D.H., Kang J.H., Kim S.H., Kwak H.B. (2018). Role of exercise in age-related sarcopenia. J. Exerc. Rehabil..

[57] Steffl M., Bohannon R.W., Sontakova L., Tufano J.J., Shiells K., Holmerova I. (2017). Relationship between sarcopenia and physical activity in older people: A systematic review and meta-analysis. Clin. Interv. Aging.

[58] Casey P.H., Goolsby S.L., Lensing S.Y., Perloff B.P., Bogle M.L. (1999). The use of telephone interview methodology to obtain 24-hour dietary recalls. J. Am. Diet. Assoc..

[59] Cudjoe T.K.M., Kotwal A.A. (2020). “Social Distancing”Amid a Crisis in Social Isolation and Loneliness. J. Am. Geriatr. Soc..

